# Can the breast screening appointment be used to provide risk assessment and prevention advice?

**DOI:** 10.1186/s13058-015-0595-y

**Published:** 2015-07-09

**Authors:** D. Gareth Evans, Anthony Howell

**Affiliations:** Genesis Breast Cancer Prevention Centre, University Hospital of South Manchester NHS Trust, Southmoor Road, Wythenshawe, Manchester M23 9LT UK; Genomic Medicine, Manchester Academic Health Science Centre, University of Manchester, Central Manchester Foundation Trust, St. Mary’s Hospital, Oxford Road, Manchester, M13 9WL UK; Manchester Breast Centre, Manchester Cancer Research Centre, University of Manchester, Christie Hospital, Wilmslow Road, Withington, Manchester M20 4BX UK

## Abstract

Breast cancer risk is continuing to increase across all societies with rates in countries with traditionally lower risks catching up with the higher rates in the Western world. Although cure rates from breast cancer have continued to improve such that absolute numbers of breast cancer deaths have dropped in many countries despite rising incidence, only some of this can be ascribed to screening with mammography, and debates over the true value of population-based screening continue. As such, enthusiasm for risk-stratified screening is gaining momentum. Guidelines in a number of countries already suggest more frequent screening in certain higher-risk (particularly, familial) groups, but this could be extended to assessing risks across the population. A number of studies have assessed breast cancer risk by using risk algorithms such as the Gail model, Tyrer-Cuzick, and BOADICEA (Breast and Ovarian Analysis of Disease Incidence and Carrier Estimation Algorithm), but the real questions are when and where such an assessment should take place. Emerging evidence from the PROCAS (Predicting Risk Of Cancer At Screening) study is showing not only that it is feasible to undertake risk assessment at the population screening appointment but that this assessment could allow reduction of screening in lower-risk groups in many countries to 3-yearly screening by using mammographic density-adjusted breast cancer risk.

## Introduction

Breast cancer (BC) is a major burden to society and individuals, with 49,936 women developing the disease in the UK (population of 60 million) in 2011 and 11,684 dying [1]. Although deaths from BC have been decreasing in many Western countries, the incidence of the disease continues to increase. In particular, in countries with historically low incidence, BC rates are rising rapidly, making it now the world’s most prevalent cancer. The increase in incidence is likely at least partly related to changes in weight and reproductive patterns associated with Western lifestyle. Indeed, there is evidence from genetic studies in the US, Iceland, and the UK [2–4] of a more-than-threefold increase in age-related incidence of BC not only in the general population but also in those at the highest level of risk with *BRCA1* and *BRCA2* mutations in the last 80 years [4]. BC rates are also rising in women who have not generally been screened, such as those in their forties [1]. Although cure rates continue to improve such that around 75 % of women are now cured in Western Europe and North America, the cost of treating BC is considerable, for both the women concerned and health services. Therefore, there is a need not only to predict which women in the whole population will develop the disease but also to apply drug and lifestyle measures in order to prevent the disease. BC risk assessment would be essential if risk-stratified screening and preventive measures were to be introduced on a population basis.

Currently, women 47 to 73 years of age are invited for breast screening with 3-yearly mammography in the UK. A similar approach is used in many European countries and in North America, although 2-yearly screening is the norm. A recent review of the UK National Health Service Breast Screening Programme (NHSBSP) estimated that it saved around 1,300 lives annually [5]. Although the vast majority of women in the UK receive only 3-yearly screening invitations, more frequent screening has been recommended by National Institute of Health and Care Excellence (NICE) [6] since 2004 for those at increased familial risk. This includes annual mammography at 40 to 9 years for women at moderate risk and annual screening at 40 to 60 years in those at high risk; annual magnetic resonance imaging (MRI) is recommended at 30 to 50 years in mutation carriers and those with at least a 30 % chance of a mutation in *BRCA1/2* or *TP53*. This guidance has just been updated, and the high-risk recommendations are already being implemented in the NHSBSP as highlighted recently [7]. Recent evidence suggests that stratification of risk and screening frequency is likely to be more cost-effective [8–10]. A substantial amount of BC is preventable, but chemoprevention has thus far not been applied to moderate/high-risk UK women outside randomised trials. Chemoprevention involves 5 years of the selective oestrogen receptor modulators (SERMs) tamoxifen or raloxifene, which reduce risk by 35 % to 40 % [11–13]. NICE have reviewed chemoprevention for women at moderate/high familial BC risk (at least 17 % lifetime). Final guidance is ‘offering/considering’ tamoxifen/raloxifene for women at high/moderate risk [13, 14]. However, applying such guidance requires estimation of risk in the general population of women [15, 16]. (Key NICE recommendations are summarised in Table 1).

**Table 1:** Key NICE guideline recommendations relating to risk-based early detection and prevention strategies

Early detection and prevention strategies	Moderate risk Lifetime risk 17 % to 29 %	High risk Lifetime risk 30 % + 10-year risk 8 %+	Gene carriers and 30-50 % likelihood for *TP53*, *BRCA1*, or *BRCA2*
	10-year risk 3 % to 7.9 % from age 40 to 49 years		
Mammography	Annual from age 40 to 49	Annual from age 40 to 59	Annual from age 40 to 69 except *TP53*
Magnetic resonance imaging	N/A	N/A	Annual from age 30 to 49, 25 to 59 for *TP53*
Tamoxifen	Consider 5 years of 20 mg daily	Offer 5 years of 20 mg daily	Offer 5 years of 20 mg daily
Raloxifene (post-menopausal)	Consider 5 years of 60 mg daily	Offer 5 years of 60 mg daily	Offer 5 years of 60 mg daily

N/A, not applicable; NICE, National Institute of Health and Care Excellence

## Previous attempts to undertake risk assessment on a population basis

There have been previous attempts to assess family history of BC in women at the population level [17, 18]. In a study using questionnaires in a general practice [17], 42.0 % of patients responded and 1.6 % were found to meet familial BC screening criteria at the time [19]. These data were based on only 196 replies in this age range, and the criteria were stricter at the time than later NICE guidance as there was a necessity for an average age of less than 60 years when there were two affected relatives. In a study predominantly concerning family history of 8,019 practice patients 35 to 69 years of age [17], only 4.8 % reported having a first-degree relative (FDR) with BC who was less than 70 years old. The general practice survey was based on nurses appraising BC family history at attendance for health checks and may be biased toward a more health-aware population. A different approach was taken by a Dutch study [20] that assessed the number of relatives of BC patients who would meet criteria for referral in The Netherlands under the older UK criteria [19]. The investigators found that 0.25 FDRs per patient (or an average of one for every four patients with BC) would have met eligibility criteria per case of BC in a series of 1,060 BC-affected women. If one assumes that 12.5 % of women develop BC in their lifetime [1], this might translate to 3.1 % (that is, 12.5 × 0.25) of the population. As such, family history alone is a blunt tool in a population setting and overall has poor prediction of which women will develop BC as the majority of women even with early-onset BC do not have a family history of the disease [21].

## Which risk model should be used?

A number of BC risk models have been developed in the last 25 years [22]. These incorporate known genetic, reproductive, and other risk factors to a greater or lesser extent (Table 1). Age and gender alone are extremely strong predictors of BC risk. Gail and colleagues [23, 24] described a risk assessment model which focuses primarily on non-genetic risk factors with limited information on family history. A model of relative risks for various combinations of the utilised risk factors (Table 2) was developed from case-control data from the Breast Cancer Detection Demonstration Project. Individualized BC probabilities from information on relative risks and the baseline hazard rate are generated. These calculations take competing risks and the interval of risk into account. The data depend on having periodic breast surveillance. The Gail model was originally designed to determine eligibility for the Breast Cancer Prevention Trial [23] and has since been modified (in part to adjust for race) and made available on the National Cancer Institute website [25]. The model has been validated in a number of settings and probably works best in general assessment clinics, where family history is not the main reason for referral [23–25], but it should also be useful for utilization in general population screening programmes, as it has now been recalibrated to reflect more recent BC incidence rates. The major limitation of the Gail model is the inclusion of only first-degree relatives, which results in underestimating risk in the 50 % of the group with familial risk of cancer in the paternal lineage. The model also takes no account of age of onset of BC.

**Table 2:** Known breast cancer risk factors and their incorporation into existing risk prediction models

	Relative risk at extremes	Gail	Claus	BRCAPRO Ford	Tyrer-Cuzick	BOADICEA
Prediction						
Personal information						
Age (20 to 70)	30 (20 versus 70)	Yes	Yes	Yes	Yes	Yes
Body mass index/weight gain	2 (loss versus gain from 30) [49]	No	No	No	Yes	No
Alcohol intake	1.28 [28] (0 versus 4 units) daily	No	No	No	No	No
Hormonal/reproductive factors						
Age of menarche	1.5 (<10 versus >16) [57]	Yes	No	No	Yes	No
Age of first live birth	3 (>35 versus <19) [58]	Yes	No	No	Yes	No
Age of menopause	2 (>55 versus <40) [57]	No	No	No	Yes	No
Hormone replacement therapy use	2 (combined for 10 years current versus never) [59]	No	No	No	Yes	No
Oral contraceptive pill use	1.24 (current versus never) [60]	No	No	No	No	No
Breast feeding	0.8 (>4 years versus none) [61]	No	No	No	No	No
Plasma oestrogen	6 [62]	No	No	No	No	No
Personal breast disease						
Breast biopsies	2 [63]	Yes	No	No	Yes	No
Atypical ductal hyperplasia	4 [63]	Yes	No	No	Yes	No
Lobular carcinoma *in situ*	4 [63]	No	No	No	Yes	No
Mammographic breast density	6 [64]	Has been modelled in some studies	No	No	Yes	No
Family history						
First-degree relatives	3.6 [65] (2 versus none in FDR)	Yes	Yes	Yes	Yes	Yes
Second degree relatives	1.5 [65]	No	Yes	Yes	Yes	Yes
Third-degree relatives		No	No	No	No	Yes
Age of onset of breast cancer	3 (<50 in sister versus none) [65]	No	Yes	Yes	Yes	Yes
Bilateral breast cancer	3 (<50 in FDR versus none)	No	No	Yes	Yes	Yes
Ovarian cancer	1.5 [26]	No	No	Yes	Yes	Yes
Male breast cancer	3 (<45 years in daughters) [64]	No	No	Yes	Yes	Yes
Genetic testing						
*BRCA1/2*	15	No	No	Yes	Yes	Yes
SNPs	10 (top 1 % versus bottom 1 % for 77 SNPs) [67]	Has been added in some studies	No	Soon	Soon	Soon

BOADICEA, Breast and Ovarian Analysis of Disease Incidence and Carrier Estimation Algorithm; FDR, first-degree relative; SNP, single-nucleotide polymorphism. Table adapted from [68]

The Claus model [26] and BRCAPRO [27] are primarily genetic models calculating the likelihood of either a putative high-risk dominant gene [26] or *BRCA1/2* [27]. BC risks are imputed from this calculation. As such, given the rarity of *BRCA1/2* or the putative dominant gene in the Claus model, these models are useful in the familial setting only and are not relevant to assessment on a population basis in which the great majority of women have no family history. BOADICEA (Breast and Ovarian Analysis of Disease Incidence and Carrier Estimation Algorithm) [28] is another model developed primarily to assess genetic risk but has been validated in a population-based series of BCs. Although inclusion of non-genetic risks are anticipated, these are not yet available in the online model.

The Tyrer-Cuzick model [29], based partly on a dataset acquired from the International Breast Intervention Study (IBIS) and other epidemiological data, incorporates both familial and non-genetic risk factors in a comprehensive way [29]. The major advantage over the Claus model and BRCAPRO is that the model gives consideration to the presence of multiple genes of differing penetrance. It does give a read-out of *BRCA1/2* but also gives consideration to a lower-penetrance BRCAX. As such, the Tyrer-Cuzick model addresses many of the pitfalls of the previous models, significantly the combination of extensive family history, markers of endogenous oestrogen exposure, and benign breast disease (atypical hyperplasia). Therefore, it is unsurprising that the model tends to perform better than the simpler Gail model, particularly in the familial setting [22].

## Mammographic density

Mammographic density is the single assessable risk factor with the largest population-attributable risk and also has a substantial heritable component [30, 31]. The difference in risk between women with extremely dense, as opposed to predominantly fatty, breasts is approximately four- to six-fold [32]. Incorporation of mammographic density into standard risk prediction models has been associated with some improvement in precision of risk prediction [33, 34].

## Genetic variant assessment

A large number of common genetic variants have now been linked to small increases or decreases in BC risk [35]. These variants, called single-nucleotide polymorphisms (SNPs), appear to act in a multiplicative fashion. Those women with no family history of BC can potentially reach a high lifetime risk from the multiplicative effects of SNPs alone [36]. So far, 94 validated SNPs for BC risk have been published [37]. Use of a much smaller panel of SNPs did improve the Gail model in predicting BC [38] and seems to be particularly useful in women with dense breasts [39]. One study identified the proportion of the female population who are at moderate or high risk of BC and hence eligible for increased surveillance and the offer of chemoprevention as per NICE guidance [40]. This study in Canada also showed that Tyrer-Cuzick substantially outperformed the Gail model [40].

## Model validation in the general population

A study from Marin County in California [41] used data from 12,843 participants who have a higher background risk of BC. Of these women, 203 developed BC during a 5-year follow-up period, showing that Tyrer-Cuzick has an area under the curve (AUC) of 0.65 (95 % confidence interval (CI) 0.61 to 0.68) compared with 0.62 (95 % CI 0.59 to 0.66) for Gail and 0.60 (95 % CI 0.56 to 0.63) for BRCAPRO. The corresponding estimated expected-to-observed ratios for the models were 1.08 (95 % CI 0.95 to 1.25), 0.81 (95 % CI 0.71 to 0.93), and 0.59 (95 % CI 0.52 to 0.68). In women with an age at first birth of more than 30 years, the AUCs for the Tyrer-Cuzick, Gail, and BRCAPRO models were 0.69 (95 % CI 0.62 to 0.75), 0.63 (95 % CI 0.56 to 0.70), and 0.62 (95 % CI 0.56 to 0.68) and the expected-to-observed ratios were 1.15 (95 % CI 0.89 to 1.47), 0.81 (95 % CI 0.63 to 1.05), and 0.53 (95 % CI 0.41 to 0.68), respectively. A further North American study has shown that Tyrer-Cuzick substantially outperformed the Gail model [42]. The authors applied 10-year absolute risks of BC, using prospective data from 1,857 women over a mean follow-up length of 8.1 years of whom 83 developed cancer. The 10-year risks assigned by Gail and Tyrer-Cuzick differed ranging widely from 0.001 % to 79.5 %. The mean Gail and Tyrer-Cuzick assigned risks of 3.2 % and 5.5 %, respectively, were lower than the cohort’s 10-year cumulative probability of developing BC of 6.25 %. Agreement between assigned and observed risks was better for Tyrer-Cuzick (HL X4(2) = 7.2, *P* = 0.13) than for Gail, which significantly under-predicted cancers (*P* < 0.001). The Tyrer-Cuzick model also showed better discrimination (AUC = 69.5 %, CI = 63.8 % to 75.2 %) than the Gail model (AUC = 63.2 %, CI = 57.6 % to 68.9 %). In almost all covariate-specific subgroups, Gail mean risks were significantly lower than the observed risks, whereas Tyrer-Cuzick risks showed generally good agreement with observed risks, even in the subgroups of women who were considered to be at average risk (for example, who had no family history of BC or who were *BRCA1/2* mutation-negative). The AUC performances are summarised in Table 3.

**Table 3:** Performance of risk models assessed in at least two models

Risk model performance (country)	Gail	Claus	BRCAPRO Ford	Tyrer-Cuzick	BOADICEA
Quante et al. [42] (USA)	0.632 (0.576-689)	Not assessed	Not assessed	0.695 (0.638- 0.752)	Not assessed
Powell et al. [41] (USA)	0.62 (0.59-0.66)	Not assessed	0.60 (0.56-0.63)	0.65 (0.61-0.68)	Not assessed
Performance in general population	Well validated but needs more precision in familial setting	Not useful	Not useful	Has been validated and outperforms Gail	May be useful but requires addition of non-genetic factors

Percentages are area under the curve with 95 % confidence intervals. BOADICEA, Breast and Ovarian Analysis of Disease Incidence and Carrier Estimation Algorithm

The Manchester PROCAS (Predicting Risk Of Cancer At Screening) study has also shown that Tyrer-Cuzick is superior to Gail in predicting which women develop BC (Brentnall et al.; 2015; Development of a mammographic density adjusted Tyrer-Cuzick model in 50,000 women in a general population breast screening population: implications for risk stratified screening; unpublished). Importantly, adding an adjustment for mammographic density increases the identification of women at moderate (4,863, 9.9 %) and high (1,402, 2.8 %) risk. The cancer rates in these groups were three and four times higher than the lower-risk groupings. Additionally, rates of higher-stage cancers were further increased, suggesting that targeting these groups would allow prevention of more lethal BCs (Brentnall et al.; 2015; Development of a mammographic density adjusted Tyrer-Cuzick model in 50,000 women in a general population breast screening population: implications for risk stratified screening; unpublished).

## When is the best opportunity to offer risk assessment?

It is difficult to apply the NICE guidelines for additional surveillance or offer of chemoprevention within the National Health Service since there is currently no systematic mechanism for identifying moderate/high-risk women. Many/most moderate/high-risk women remain unaware of their BC risk, and only those women who present with concerns about family history are referred to family history clinics. These women are usually under 50 and are offered additional screening (every 12 to 18 months) and chemoprevention. The one occasion when all women are invited for a health-related visit that appears to be a good related opportunity is their first invitation for screening mammography. In most countries with national population-based screening programmes, this will be at around age 50.

We have undertaken a National Institute for Health Research-funded study (PROCAS) in Greater Manchester, which is investigating the feasibility of assessing and communicating BC risk to women in the NHSBSP [43]. To date, over 57,000 women have been recruited from those invited for mammography (43 % uptake). Interestingly, 95 % of recruited women indicated that they wished to know their risk of BC. Since risk is not estimated in the general population, it has not been possible until now to assess the proportion of women in the NHSBSP who are at moderate or high risk by NICE criteria or by models of risk which include factors other than family history.

We have shown that, among women entering the PROCAS population-based screening cohort study in Greater Manchester at the age of 46 to 49, 0.7 % met high-risk and 3.0 % moderate-risk criteria of the NICE algorithm [44]. Only five (13.5 %) out of 37 of the BCs would have been identified in 160 women if just these elevated risk women had been screened from age 46 years onwards [43]. The odds of BCs were 5.7 (95 % CI 1.9 to 14.7) times higher than 17 (0.6 %) out of 3,033 of those diagnosed in women with no family history of BC. These finding are at an early stage in follow-up, and more time is required to assess whether with further cancers NICE guidance on extra screening in this age group is justified [7]. The PROCAS study has highlighted a disparity between the NICE algorithm and the 10-year risk thresholds as calculated by Tyrer-Cuzick as 8.8 % of women would have qualified under the NICE 3 % 10-year risk threshold of age of 40 years [44]. This is not surprising as the NICE algorithm was set to identify women on the basis of family history alone who would usually meet the 10-year threshold. The additional numbers identified by Tyrer-Cuzick are of those who, in addition to a less significant family history, had other BC risk factors (nulliparity and late first childbirth) such as in three women who developed BC with a single FDR in their fifties [44].

When an adjustment is made to the Tyrer-Cuzick model on the basis of a radiologist’s assessment of percentage breast density on a Visual Analogue Scale, the proportion of women meeting high- and moderate-risk criteria rose to 2.8 % and 9.9 %, respectively, in the whole 50,000 population thus far assessed (Evans et al.; 2015; Identifying and informing women at high and low-risk of breast cancer in the general population: risk feedback from the first 50,000 women in the Predicting the Risk of Cancer at Screening (PROCAS) study; unpublished). This stratification accurately identified not only those at higher risk but also those with a higher incidence of high-stage (2b or 3) BCs who might have benefited from more frequent surveillance.

The PROCAS study has also already shown that it is feasible to collect questionnaire data from women when they attend the screening episode [43]. We have highlighted that certain questions such as whether a relative has had bilateral BC may need validating and would be suitable for an online questionnaire with a prompting system (Evans et al.; 2015; Identifying and informing women at high and low-risk of breast cancer in the general population: risk feedback from the first 50,000 women in the Predicting the Risk of Cancer at Screening (PROCAS) study; unpublished). The PROCAS study has now given risk feedback to nearly 700 women, and 75 % of those invited at high risk (at least 8 % 10-year risk) attended a telephone interview or face-to-face appointment. Re-attendance rates increased significantly to 94 % (229 out of 244) in high-risk women who had been invited for a follow-up 3-year mammogram and were equivalent to usual re-attendance rates in those at low risk (84 %) (Evans et al.; 2015; Identifying and informing women at high and low-risk of breast cancer in the general population: risk feedback from the first 50,000 women in the Predicting the Risk of Cancer at Screening (PROCAS) study; unpublished).

At least two other large studies with aims similar to those of PROCAS are under way. The Karma study in Sweden aims to recruit 100,000 women at their 2-yearly screening appointment [45]. Blood DNA is collected in addition to questionnaire data and mammographic density measurement [45]. PRISMA (Preferred Reporting Items for Systematic Reviews and Meta-Analyses) (Mireille Broeders, personal communication) started recruitment in The Netherlands in 2014 and aims to collect risk factor data on 90,000 screening participants, including 30,000 blood samples.

## What is the best age to undertake risk assessment?

Only 20 % of BC occurs at ages below 50 years, when most population-based screening programmes commence. However, 15 % do occur between the ages of 40 to 50 years, and women in this age group could benefit from NICE-approved extra surveillance and chemoprevention. Primary care physicians and all others undertaking risk assessment need to be aware that moderate risk can be achieved with less significant family history if there are other adverse risk factors. Around 3.7 % (95 % CI 3.1 % to 4.3 %) of women in PROCAS at 46 to 49 years of age would have met NICE moderate/high-risk criteria following the NICE algorithm at 40 years of age, but this rises to 8.8 % (95 % CI 8.0 % to 9.7 %) when Tyrer-Cuzick is used (Evans et al.; 2015; Identifying and informing women at high and low-risk of breast cancer in the general population: risk feedback from the first 50,000 women in the Predicting the Risk of Cancer at Screening (PROCAS) study; unpublished). Thus, up to 8.8 % of the female population in their forties could be offered annual mammography as per moderate-risk screening recommendations and be at least considered for tamoxifen [44]. When expanded to the whole population of PROCAS, 4,225 (8.6 %) of 49,639 women (thus far evaluated) met moderate-risk criteria, and 597 (1.2 %) met the 8 % 10-year risk high-risk criteria. Feedback has been offered to 866 women at high-risk, including those with moderate risk + >60 % (high) mammographic density (which equates to an 8 % 10-year risk), and 568 (78.5 %) of these women have received risk feedback. Two hundred and eighty-three (86 %) out of 330 of those offered extra interval screening have commenced additional breast screening. Four BCs have now been identified at an early stage on the 18-month mammogram [45].

## What preventive options should be discussed?

### Lifestyle

Women attending a risk assessment appointment could have a wide-ranging discussion on modifiable BC risks regardless of their actual predicted risk of BC. Some authors have suggested that half of BCs may be prevented by reversing the major modifiable risk factors, including achieving and maintaining a healthy weight, regular physical activity, minimal alcohol intake, and chemoprevention [46]. The risk appointment would be an opportunity to provide health advice related to weight gain, which has a large effect on BC risk as well as other cancers (especially endometrial), diabetes, and heart disease. High-quality observational data show that weight gain in the pre-menopausal period and being overweight or obese after the menopause increase BC risk [46, 47]. A meta-analysis estimated that for each 5-kg/m^2^ increase in body mass index the risk of BC was increased by 12 % [48]. Further evidence from two large observational studies indicates that pre- or post-menopausal weight loss reduces post-menopausal BC risk. Sustained weight reduction of 5 % of body weight in the IOWA Women’s Health Study reduced post-menopausal BC risk by 25 % to 40 % compared with women who continued to gain weight [49]. In the Nurses’ Health Study, post-menopausal women who maintained a body weight reduction of 10 kg or more and did not take hormone replacement therapy had a 50 % reduction in BC risk [50]. A review of 73 observational studies indicated that moderate- to vigorous-intensity physical activity reduced the risk of BC by approximately 25 % in pre- and post-menopausal women compared with inactive women [51]. Alcohol is estimated to increase BC risk by 7 % to 10 % for each 10-g increase in daily alcohol intake [35]. The Nurses’ Health Study showed that women who consumed three to six drinks per week were 15 % more likely to develop BC compared with never-drinkers [52]. On the basis of the evidence available, all women could be advised at a risk evaluation at their screening appointment to maintain a healthy weight or lose weight if overweight, to take regular exercise, and to minimize alcohol intake.

### Chemoprevention

There have been nine randomised trials of SERMs [13] and two trials of aromatase inhibitors [53, 54]. These have been carried out mainly in women at increased risk of BC, but one trial was in women with osteoporosis and another in women with or at high risk of diabetes or heart disease (raloxifene). In the SERM trials, 83,399 participants were included, with more than 300,000 years of follow-up over an average period of 65 months. The overall reduction in BC (including ductal carcinoma *in situ*) using tamoxifen 20 mg per day was 38 % (*P* < 0.0001) [13], and estimated reduction in 10-year cumulative BC incidence ranged from 6.3 % in the control group to 4.2 % in the SERM groups. On the basis of this evidence, NICE approved the use of both tamoxifen and raloxifene (post-menopausal only) for prevention of BC in women at familial risk [7]. When compared in a randomised trial, tamoxifen was significantly superior to raloxifene in longer-term follow-up for preventing invasive BC (relative risk raloxifene/tamoxifen 1:24, 95 % CI 1.05 to 1.47), but raloxifene was associated with fewer side effects, particularly with respect to the uterus, and may be preferable in post-menopausal women [14]. A recent analysis of the IBIS I tamoxifen prevention trial indicated that the reduction of risk lasted up to 15 years after the 5-year treatment with tamoxifen [55].

Aromatase inhibitors given after surgery to prevent BC relapse are generally superior to tamoxifen. This led to the initiation of two placebo-controlled trials in post-menopausal women at increased risk of BC. One tested exemestane and reported a 65 % reduction in BC risk after 5 years of treatment [53]. In the other trial (IBIS II), anastrozole was compared with placebo [54]. In total, 3,864 post-menopausal women who were between 40 and 70 years old and at increased risk of BC were randomly assigned. The first report indicated that BC incidence was reduced by 53 % (hazard risk 0.47, 95 % CI 0.32 to 0.68) by use of anastrozole. Compared with SERMs, aromatase inhibitors are not associated with an increased risk of thromboembolic disease and uterine problems, including cancer, but are associated with increased mild to moderate bone/muscle pain and reduced bone density.

Unfortunately, the IBIS II study was not available for NICE guidance and therefore aromatase inhibitor use in the UK would be without the current backing of NICE. Clearly, for chemoprevention to have a major impact on BC incidence, high levels of uptake would be required although potentially more than 20 % of BCs could be targeted [44].

## Conclusions

The first mammography screening appointment at 47 to 50 years of age provides an ideal teachable moment to assess BC risk and inform women of lifestyle and other measures to reduce BC risk. In the future, this may also allow the introduction of risk-stratified screening which already exists to some extent in many screening programmes; the NHSBSP has already introduced very high-risk screening with MRI mainly in women with mutations in *BRCA1*, *BRCA2*, or *TP53*. The PROCAS study has shown that it is feasible to collect risk information and, using a good discriminating risk model, provide accurate assessments of women’s risk of BC, although further improvements in risk discrimination are needed. These models should ideally be adapted to include mammographic density information and potentially common genetic risk modifiers (SNPs). The screening appointment would provide an opportunity to help reduce disease burden from not only BC but other common diseases. It appears that the great majority of women wish to know their risk, although this would need to be validated by inviting whole populations to an opt-in or -out risk assessment rather than seeking consent for a research study. More widespread introduction of chemoprevention, using tamoxifen in pre-menopausal women and raloxifene or aromatase inhibitors in post-menopausal women, could substantially reduce BC incidence, although uptake rates would need to rise above the approximately 10 % seen in most studies [56]. Further research would be required to determine whether women need to be reassessed at least at one further time point to determine changes to risk related to menopausal status, mammographic density, and other changes to risk factors. An assessment of whether to introduce a single appointment at age 40 to plan future screening would also be desirable.

## Abbreviations

AUC: Area under the curve
BC: Breast cancer
CI: Confidence interval
FDR: First-degree relative
IBIS: International Breast Intervention Study
MRI: Magnetic resonance imaging
NHSBSP: National Health Service Breast Screening Programme
NICE: National Institute of Health and Care Excellence
PROCAS: Predicting Risk Of Cancer At Screening
SERM: Selective oestrogen receptor modulator
SNP: Single-nucleotide polymorphism
